# Motor Sequence Learning Is Associated With Hippocampal Subfield Volume in Humans With Medial Temporal Lobe Epilepsy

**DOI:** 10.3389/fnhum.2018.00367

**Published:** 2018-09-26

**Authors:** Jinyi Long, Yanyun Feng, HongPeng Liao, Quan Zhou, M. A. Urbin

**Affiliations:** ^1^College of Information Science and Technology, Jinan University, Guangzhou, China; ^2^Department of Radiology, The First People’s Hospital of Foshan, Foshan, China; ^3^School of Automation Science and Engineering, South China University of Technology, Guangzhou, China; ^4^Department of Neurology, The First People’s Hospital of Foshan, Foshan, China; ^5^Department of Physical Medicine and Rehabilitation, University of Pittsburgh, Pittsburgh, PA, United States

**Keywords:** epilepsy, motor sequence learning, medial temporal lobe, hippocampus, MRI

## Abstract

**Objectives**: Medial temporal lobe epilepsy (mTLE) is characterized by decreased hippocampal volume, which results in motor memory consolidation impairments. However, the extent to which motor memory acquisition are affected in humans with mTLE remains poorly understood. We therefore examined the extent to which learning of a motor tapping sequence task is affected by mTLE.

**Methods**: MRI volumetric analysis was performed using a T1-weighted three-dimensional gradient echo sequence in 15 patients with right mTLE and 15 control subjects. Subjects trained on a motor sequence tapping task with the left hand in right mTLE and non-dominant hand in neurologically-intact controls.

**Results**: The number of correct sequences performed by the mTLE patient group increased after training, albeit to a lesser extent than the control group. Although hippocampal subfield volume was reduced in mTLE relative to controls, no differences were observed in the volumes of other brain areas including thalamus, caudate, putamen and amygdala. Correlations between hippocampal subfield volumes and the change in pre- to post-training performance indicated that the volume of hippocampal subfield CA2–3 was associated with motor sequence learning in patients with mTLE.

**Significance**: These results provide evidence that individuals with mTLE exhibit learning on a motor sequence task. Learning is linked to the volume of hippocampal subfield CA2–3, supporting a role of the hippocampus in motor memory acquisition.

**Highlights**

- Humans with mTLE exhibit learning on a motor tapping sequence task but not to the same extent as neurologically-intact controls.
- Hippocampal subfield volumes are significantly reduced after mTLE. Surrounding brain area volumes do not show abnormalities.
- Hippocampal subfield CA2–3 volume is associated with motor sequence learning in humans with mTLE.

## Introduction

The hippocampus is involved in procedural memory, a type of memory necessary for motor sequence learning (Albouy et al., 2013). During a serial reaction time task, amnesic patients have been shown to outperform controls by repeating the sequence of key presses to the location of the stimulus through increasingly rapid performance. However, these same patients exhibit an impaired ability to recognize the sequence (Reber and Squire, 1998). Medial temporal lobe epilepsy (mTLE) is characterized by seizures originating in mesial temporal lobe structures. Decreased hippocampal volume ipsilateral to the epileptogenic temporal lobe has been reported in this patient population (Marsh et al., 1997). At present, it is not known whether motor sequence learning is preserved in humans with mTLE.

Neural correlates of motor sequence learning have been characterized in neurologically-intact controls and include the cerebellum, basal ganglia, supplementary motor area, as well as primary motor and premotor cortices (Willingham et al., 2002; Doyon et al., 2003). However, the role of hippocampus during motor sequence learning is still controversial. Most prior work has not implicated the hippocampus in motor sequence learning (Curran, 1997; Clark and Squire, 1998; Chun and Phelps, 1999; Poldrack et al., 2001), but other findings suggest that the hippocampus is necessary irrespective of whether knowledge of the sequence was implicitly or explicitly acquired (Grafton et al., 1995; Schendan et al., 2003). The hippocampus is thought to support motor sequence learning by encoding temporally discontiguous but structured information and events (Schendan et al., 2003; Eichenbaum, 2004; Albouy et al., 2008).

Hippocampus is composed of several histologically distinct subfields: subiculum, cornu ammonis sectors (CA)1–4, and dentate gyrus (DG). There is evidence that histological differences influence functional characteristics of each subfield. Animal studies suggest a selective role for CA1 pyramidal cells in intermediate and long-terms patial learning or memory consolidation, but not in short-term acquisition or encoding (Blum et al., 1999; Remondes and Schuman, 2004; Vago et al., 2007). Rather, CA2–3 is thought to be responsible for encoding and early retrieval (Hasselmo, 2005; Acsády and Káli, 2007). In addition, there is evidence that CA1 pyramidal cells are less critically involved in declarative memory compared to DG granule cells or CA4 pyramidal cells in humans with TLE (Coras et al., 2014).

The goal of our study was to examine the effect of mTLE on motor sequence learning. Considering the spatial recall demands of a motor tapping sequence, we hypothesized that mTLE patients will exhibit a reduced ability to learn a motor sequence task. To test this hypothesis, 15 patients with right mTLE and 15 controls trained on a motor sequence tapping task, and MRI volumetric analysis was performed using a T1-weighted three-dimensional gradient echo sequence.

## Materials and Methods

### Subjects

We recruited patients with unilateral mTLE from the First People’s Hospital of Foshan. First, each patient underwent non-invasive neurophysiologic evaluation via interictal EEG recordings and extensive video-EEG monitoring to record seizures. Next, two examiners independently obtained the evaluation of ictal semiology and defined the cerebral structures impacted by epileptic activity according to clinical and EEG features of the seizures. In addition, 3-T MRI was performed to investigate temporal lobe structures in detail for all patients. The presence of medial temporal sclerosis was evaluated qualitatively by visual inspection of structure MRI. Raters were blind to motor sequence learning results. Patients with epileptic paroxysms in extra-temporal regions on EEG were excluded. A total of 15 mTLE patients (seven males, 29.9 ± 7.8 years of age) with right (onset) unilateral seizures were enrolled. Demographics and clinical information of patients are provided in Table 1. All subjects with TLE exhibited MRI evidence of right hippocampal sclerosis. We also recruited 15 healthy adults (nine males, 29.1 ± 9.1 years of age) to serve as controls. Musicians were excluded from the sample. All subjects were right-handed. All subjects gave their written informed consent prior to the study, which was in accordance with the Declaration of Helsinki and approved by the local ethics committee at the Jinan University and the First People’s Hospital of Foshan.

**Table 1:** Demographic and clinical characteristics (Mean ± SD).

	mTLE	Control	*p*-value
Sample size	15	15	—
Sex (male/female)	7/8	9/6	0.74^a^
Age (years)	29.9 ± 7.8	29.1 ± 9.1	0.67^b^
Education (years)	11.2 ± 3.8	13.6 ± 3.5	0.51^b^
Epilepsy duration (years)	12.5 ± 10.8	—	
Age at epilepsy onset	10.7 ± 9.1	—	
Frequency (times/year)	22.3 ± 31.7	—	

*^a^Chi-square test; ^b^two-sample t-test*.

### MRI Data Acquisition

A 3-T MR imaging system (General Electric) was used for scanning. A high resolution three-dimensional T1-weighted image was acquired via a 3D-fast spoiled gradient recall sequence with the following parameters: IR = 450 ms, flip angle = 15°, and FOV = 24 × 24 cm^2^. One hundred and forty-six slices with a slice thickness of 1 mm were acquired to construct a 256 × 228 data matrix.

### Structural Volume Evaluation

The volumetric segmentation was performed with experimental software (Freesurfer package v5.1^1^), which provided fully automatic cortical parcellation and segmentation of subcortical structures. The program calculates brain sub-volumes by assigning a neuro-anatomical label to each voxel based on probabilistic information estimated automatically from a manually labeled training set. Briefly, this process includes motion correction, removal of non-brain tissue using a hybrid watershed/surface deformation procedure, multiple intensity and spatial normalization, Talairach transformation, segmentation of the subcortical white matter and deep gray matter structures (Fisch et al., 2004; Ségonne et al., 2004). Details regarding the process and analysis pipeline has been described elsewhere^1^. Finally, 12 hippocampal sub-regions in the left or right hemisphere were automatically obtained: fimbria, CA1, CA2-CA3, CA4-DG, subiculum and presubiculum. All sub-regions from each participant were visually inspected to detect visible errors in segmentation. The whole hippocampus volume was obtained by adding all hippocampal subfields. In addition to hippocampal subfields, we also calculated the volumes of surrounding brain areas that served as references for volumes outside of the hippocampus. These reference areas included thalamus, caudate, putamen and amygdala in the right hemisphere. The total intracranial volume (TIV) was also automatically calculated by FreeSurfer software. Volumes of the hippocampal subfields and surrounding brain areas were adjusted for TIV using the following formula (Buckner et al., 2004):

(1) $${\text{Volume}}_{\text{adj}}{\text{=Volume}}_{\text{observed}}-\beta \left({\text{TIV}}_{\text{observed}}-{\text{TIV}}_{\text{sample mean}}\right)$$

where, *β* is the slope of the regional volume regression on TIV_observed_.

The volumetric segmentation was also performed with experimental software of Freesurfer package v6.0 (Iglesias et al., 2015). Finally, 12 hippocampal sub-regions in the left or right hemisphere were automatically obtained including hippocampal tail, subiculum, CA1, hippocampal fissure, presubiculum, parasubiculum, molecular layer, granule cell layer of DG (GC-DG), CA2-CA3, CA4, fimbria and hippocampus-amygda-la-transition-area (HATA). The TIV was also automatically calculated. Volumes of the hippocampal subfields were adjusted for TIV using the formula as described above.

### Motor Sequence Learning Task

**Figure 1:**
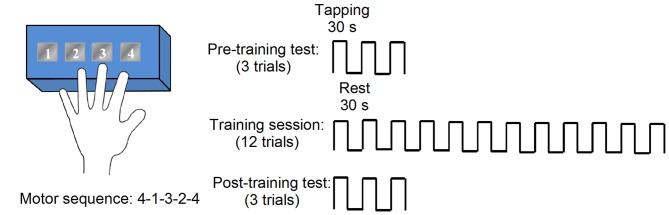
Motor sequence task consisted of blocks of multiple trials, with each trial consisting of an active tapping period (30 s) and a rest period (30 s). Tapping periods required subjects to repeatedly tap a five-element numerical sequence (e.g., 4-1-3-2-4) on a numbered button box. Pre- and post-training performance tests were comprised of three trials, while training involved 12 trials.

Subjects performed a finger-tapping task in a particular sequence during a pre-training performance test, a training protocol, and a post-training performance test (Figure 1; Korman et al., 2003; Walker et al., 2003). During motor sequence learning task, the seizures were not monitored. Note that the post-test was performed immediately following training on the motor sequence. T1 images were acquired upon enrollment in the study and before behavioral testing. Participants were instructed to press four numeric keys on a standard computer keyboard with the fingers of the left-hand in right mTLE and finger of the non-dominant, left hand in controls. Each trial of the task involved repeating the same five-element sequence (4-1-3-2-4) as quickly and accurately as possible during a 30-s interval, followed by 30 s of rest. Subjects were instructed to not correct for errors and to continue tapping without pause as smoothly as possible. During a trial, the sequence was displayed on a monitor in front of the subject. Each key press produced a dot on the monitor. Pre- and post-training tests consisted of three, 30-s trials with 30-s rest periods between each trial. Performance was measured as the total number of correct sequences completed and the number of errors in a trial. The training protocol consisted of 12, 30-s trials with 30-s rest periods between trials, lasting a total of 12 min.

### Statistical Analysis

Normal distribution was tested by the Shapiro–Wilk’s test and Mauchly’s test was used to test for sphericity. Data were log transformed when not normally distributed. When sphericity could not be assumed, the Greenhouse–Geisser correction statistic was used. Two-way analysis of variance (ANOVA) was performed to determine the effect of GROUP (mTLE and controls) and TIME (pre-training and post-training) on the numbers of errors and correct sequences. One-way ANOVAs were performed to determine the effect of GROUP on the volume size of hippocampal subfields and reference brain areas (i.e., thalamus, caudate, putamen and amygdala). In the above ANOVA analysis, age, gender and years of education (log transformed) were modeled as nuisance variables. Bonferroni *post hoc* analysis was used to test for pairwise comparisons. Partial correlation analyses (corrected for log of years of education) were used to assess the relative importance of each subfield within left and right hippocampus in predicting motor sequence learning. The threshold for significance was set at *P* < 0.008 (Bonferroni correction based on six hippocampal subfields) to control for multiple comparisons. To test the possible impact of epilepsy history and disease load (age at onset, duration, epilepsy frequency) on volume size of hippocampal subfields and motor sequence learning, nonparametric statistics (Spearman correlations) were used. We also examined the possible impact of the time between seizures and the moments of behavioral testing on motor sequence learning with spearman correlations. Significance was set at *P* < 0.05. Group data are presented as mean ± SD in the text.

## Results

### Behavioral Results

**Figure 2:**
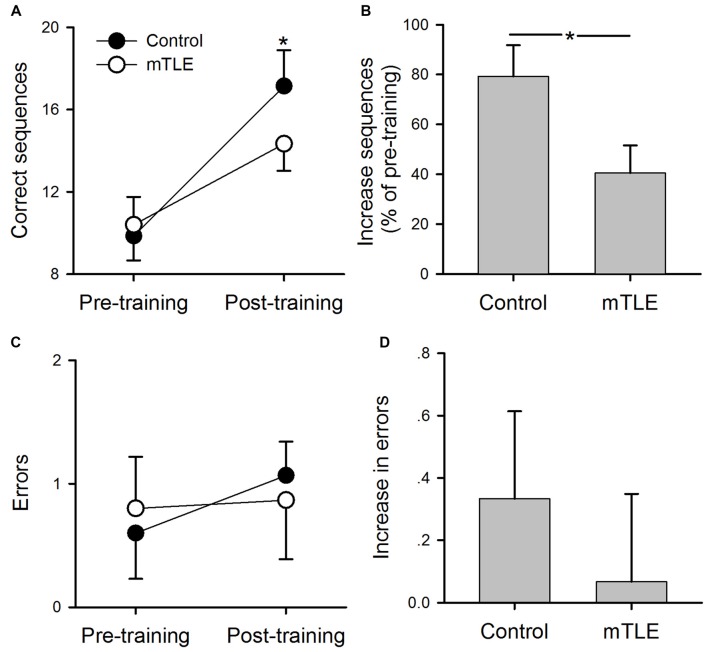
Performance on the motor sequence task. **(A)** Mean number of correct sequences completed during pre- and post-training sessions in control and medial temporal lobe epilepsy (mTLE) groups. **(B)** Percent increase in correct sequences completed from pre- to post-training in control and mTLE groups. **(C)** Mean number of errors during pre- and post-training sessions in control and mTLE groups. **(D)** Percent increase in errors from pre- to post-training sessions in control and mTLE groups. Error bars indicate SDs. *P < 0.05.

Figure 2 illustrates pre- and post-training performance (numbers of errors and correct sequences completed) during the motor sequence tapping task in mTLE and control groups. A two-way ANOVA showed a significant effect of GROUP (*F*_(1,56)_ = 21.3, *P* < 0.001), TIME (*F*_(1,56)_ = 12.4, *P* < 0.001) and in their interaction (*F*_(1,56)_ = 10.1, *P* < 0.001) on the number of correct sequences completed (Figure 2A). *Post hoc* tests showed that the number of correct sequences was greater at post-training compared with pre-training in control (*p* < 0.01) and mTLE groups (*p* < 0.01). However, the increase in the number of correct sequences was greater in controls relative to the mTLE group (*p* < 0.001, Figure 2B). There was no difference of the baseline performance between groups (*p* > 0.05). A two-way ANOVA showed no significant effect of GROUP (*F*_(1,56)_ = 1.2, *P* = 0.13), TIME (*F*_(1,56)_ = 0.85, *P* = 0.32) and in their interaction (*F*_(1,56)_ = 1.8, *P* = 0.11) on the number of errors (Figures 2C,D).

### Volumetric Results

**Figure 3:**
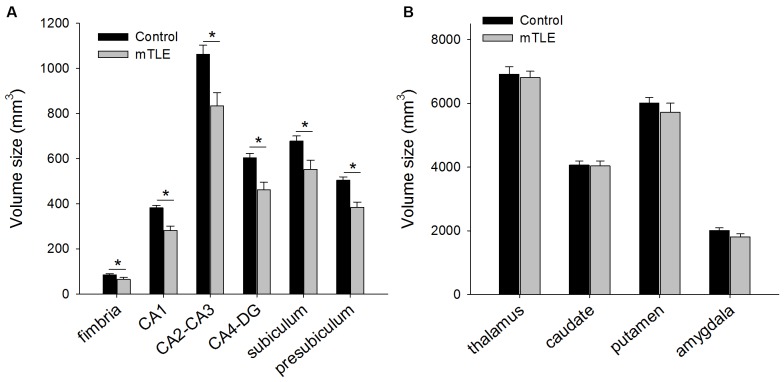
**(A)** Mean volume size of hippocampus subfields and **(B)** reference brain areas in control and mTLE groups. Error bars indicate SDs. *P < 0.05.

Figure 3 shows volume size of hippocampal subfields (Figure 3A) and reference brain areas (i.e., thalamus, caudate, putamen and amygdala; Figure 3B) in mTLE and control groups by FreeSurfer v5.1. The ANOVA for hippocampal subfields and reference brain areas showed a significant effect of GROUP on volume size (*F*_(1,19)_ = 15.23, *P* = 0.006). *Post hoc* tests indicated that volume size of all hippocampal subfields was less in the mTLE group compared to the control group: fimbria (*F*_(1,15)_ = 11.7, *P* < 0.001), CA1 (*F*_(1,15)_ = 12.4, *P* < 0.001), CA2-CA3 (*F*_(1,15)_ = 15.3, *P* < 0.001), CA4-DG (*F*_(1,15)_ = 8.1, *P* < 0.001), subiculum (*F*_(1,15)_ = 7.8, *P* < 0.001), and presubiculum (*F*_(1,15)_ = 10.5, *P* < 0.001). However, volume size of specific reference brain areas was not different between groups: thalamus (*F*_(1,15)_ = 0.61, *P* = 0.42), caudate (*F*_(1,15)_ = 0.35, *P* = 0.56), putamen (*F*_(1,15)_ = 1.5, *P* = 0.26) and amygdala (*F*_(1,15)_ = 2.01, *P* = 0.18).

Table 2 presents volume size of hippocampal subfields in mTLE and control groups by FreeSurfer v6.0. The ANOVA for hippocampal subfields in the right hemisphere showed a significant effect of GROUP on volume size (*F*_(1,11)_ = 20.1, *P* < 0.001). *Post hoc* tests indicated that volume size of all hippocampal subfields but the hippocampal fissure in the right hemisphere was less in the mTLE group compared to the control group. In addition, volume size of the hippocampal subfields in the left hemisphere was not different between groups.

**Table 2:** Hippocampal subfield volumes calculated by FreeSurfer ver.6.0 (Mean ± SD).

	mTLE (*n* = 15)	Control (*n* = 15)	*p*-value
**Left hemisphere**			
Hippocampal_tail	568.6 ± 53.2	587.1 ± 71.8	0.38
Subiculum	431.4 ± 52.3	446.3 ± 46.7	0.62
CA1	667.2 ± 75.8	681.9 ± 67.2	0.49
Presubiculum	302.7 ± 22.5	316.8 ± 36.7	0.54
Parasubiculum	52.9 ± 10.8	55.7 ± 13.1	0.68
Molecular_layer	556.3 ± 51.2	556.3 ± 51.2	0.42
GC-DG	310.8 ± 39.2	318.2 ± 35.7	0.48
CA2-CA3	195.8 ± 26.7	203.4 ± 22.4	0.71
CA4	254.9 ± 30.6	261.4 ± 27.8	0.68
Fimbria	93.3 ± 23.8	106.7 ± 27.2	0.52
HATA	61.5 ± 8.3	60.6 ± 10.6	0.76
Hippocampal_fissure	151.4 ± 25.9	156.1 ± 28.7	0.63
**Right hemisphere**			
Hippocampal_tail	391.7 ± 90.1	562.3 ± 64.9	0.38
Subiculum	305.8 ± 56.4	452.1 ± 49.5	0.62
CA1	462.3 ± 86.4	667.8 ± 62.1	0.49
Presubiculum	215.3 ± 30.6	320.1 ± 28.9	0.54
Parasubiculum	40.2 ± 8.5	58.3 ± 11.4	0.68
Molecular_layer	382.4 ± 49.1	571.3 ± 52.3	0.42
GC-DG	205.3 ± 34.5	305.7 ± 37.1	0.48
CA2-CA3	126.7 ± 27.1	221.8 ± 32.8	0.71
CA4	162.7 ± 33.2	278.1 ± 30.2	0.68
Fimbria	59.6 ± 18.2	95.5 ± 20.4	0.52
HATA	51.7 ± 10.2	61.5 ± 9.2	0.76
Hippocampal_fissure	141.7 ± 28.3	150.2 ± 23.6	0.63

### Behavior-Volumetric Correlations

**Figure 4:**
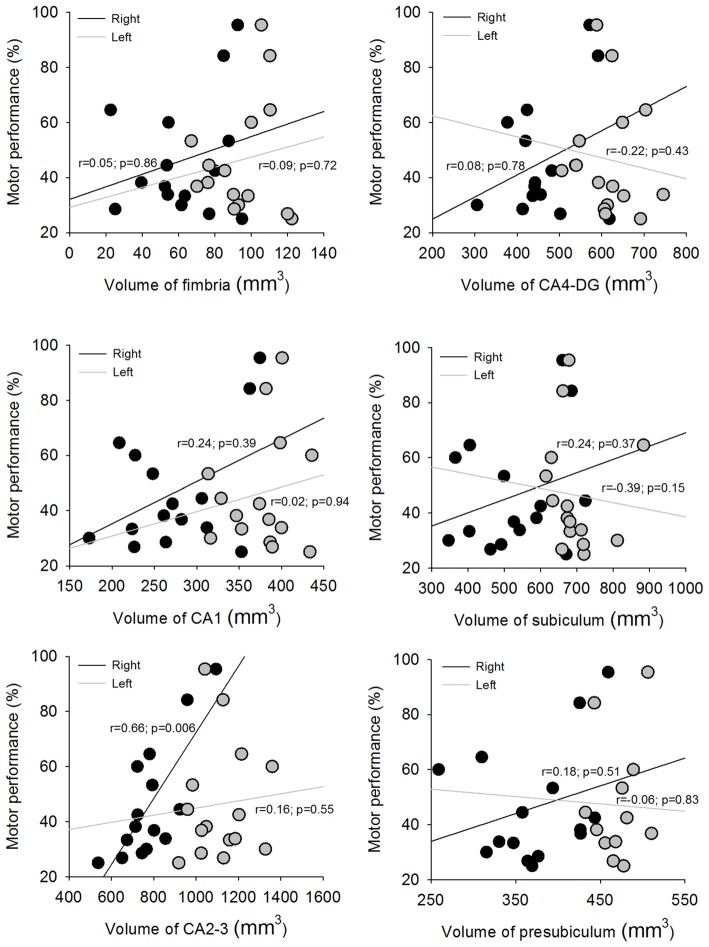
Scatter plots depicting the association between motor sequence learning and the hippocampus subfields in right and left hemispheres.

Figure 4 illustrates the association between motor sequence learning (i.e., the percentage increase of correct sequences post-training relative to pre-training) and hippocampal subfield volumes in mTLE subjects by FreeSurfer v5.1. Right hippocampal subfield CA2–3 volume was significantly correlated with motor sequence learning in mTLE subjects (*p* < 0.008; Figure 4). No other right hippocampal subfield volumes were correlated with motor sequence learning. Right hippocampal subfield volumes were not correlated with motor tapping performance at either pre-training or post-training (*p* > 0.05) nor with seizure frequency, duration of epilepsy, and epilepsy onset (*p* > 0.05). In addition, left hippocampal subfield volumes were not correlated with motor sequence learning (*p* > 0.05). Seizure history and seizure load were not correlated with motor sequence learning (*p* > 0.05). The time between seizures and the moments of behavioral testing were also not correlated with motor sequence learning (*p* > 0.05).

To test the reproducibility of our results, we also calculated the association between motor sequence learning and both sides of hippocampal subfield volumes in mTLE subjects by FreeSurfer v6. The results were similar with that using FreeSurfer v5.1. Only right hippocampal subfield CA2–3 volume was significantly correlated with motor sequence learning in mTLE subjects (*p* < 0.001). No other right or left hippocampal subfield volumes were correlated with motor sequence learning (*p* > 0.05).

## Discussion

The current study investigated the effect of mTLE on motor sequence learning. Findings indicate that patients with mTLE can learn a motor sequence tapping task, albeit to a lesser extent than neurologically-intact controls. Although all hippocampal subfield volumes were decreased in mTLE patients, CA2–3 volume was associated with motor sequence learning. Thus, results demonstrate that patients with mTLE have impairments in motor memory acquisition. However, these impairments do not preclude some degree of motor sequence learning, which is associated with the volume of hippocampal subfield CA2–3.

### Contributions of Hippocampus to Motor Sequence Learning

Consistent with previous work, we found that performance increased after training on a sequence tapping task (Korman et al., 2003; Walker et al., 2003). Since hippocampus is thought to be involved in motor sequence learning (Albouy et al., 2013), it is plausible that structural abnormalities influence learning of such a task even after practice. Previous work has shown that hippocampus is critical for encoding temporally discontiguous but structured information and events (Schendan et al., 2003; Eichenbaum, 2004). If hippocampus contributes to motor sequence learning processes, then disease states such as mTLE would likely alter neuronal interactions critical for learning in this regard. It was therefore unexpected that mTLE patients exhibited sequence learning. The percentage change in performance, however, was significantly reduced relative to controls, suggesting that learning was adversely impacted by hippocampal abnormalities. It should be noted that baseline performance was not different than controls (Figure 2A), indicating that individuals in the mTLE group did not have impairments in motor function or were otherwise unable to perform at the same level as participants in the control group. These findings align with previous work showing that amnesic patients outperform controls through intensive training on a serial reaction time task but show an impaired ability to explicitly recognize the sequence of stimuli location (Reber and Squire, 1998). Taken together, motor memory acquisition appears to be supported by medial temporal lobe structures.

### Volume Size in Hippocampal Subfields and Surrounding Brain Areas

mTLE patients had reduced hippocampal subfield volumes, a finding that is consistent with postmortem examination and other cross-sectional studies of this population (Duncan, 1997; Keller and Roberts, 2008; Mueller et al., 2012). The lack of unchanged volumes in other areas such as caudate, putamen, and amygdala for mTLE are generally in agreement previous work (Bernasconi et al., 2004; Keller and Roberts, 2008). However, patients in this study did not exhibit reductions in volume size of the thalamus, which runs counter to prior findings (Bernasconi et al., 2004). The discrepancy may be because mTLE patients in the current study did not have a history of febrile convulsions (Dreifuss et al., 2001).

### CA2–3 Contributions to Motor Sequence Learning

We found that subfield CA2–3 was the only hippocampal subfield associated with motor sequence learning in the mTLE group. There is evidence showing that sub-region CA3 plays a significant role in short-term spatial memory acquisition and encoding processes, while sub-region CA1 contributes to intermediate/long-term spatial memory and consolidation (O’Reilly and McClelland, 1994; Treves and Rolls, 1994; Kesner et al., 2004; Remondes and Schuman, 2004; Daumas et al., 2005). Within a given day, encoding of information acquired in a Hebb-Williams maze or in contextual fear conditioning is impaired by targeted lesions to the CA3 and DG sub-regions, but not from targeted lesions to the CA1 sub-region. In contrast, retention and retrieval is disrupted following lesions to CA1 across days, but not following lesions to CA3 or DG (Lee and Kesner, 2004a,b; Jerman et al., 2006). Impairments have also been demonstrated in delay-dependent retrieval without impairing immediate recall or encoding of spatial information after infusing glutamatergic antagonists (Lee and Kesner, 2002), or cyclooxygenase-2 inhibitors (Sharifzadeh et al., 2006) into the CA1 sub-region, but not when infused into the CA3 sub-region. Findings from the current study are in agreement with those of previous work and demonstrate that the integrity of the CA2–3 hippocampal subfield was correlated with motor sequence learning.

### Limitations

Aside from a small sample size, there are at least four important limitations of our study. First, the current cross-sectional study is limited in that it does not capture long-term hippocampal volume loss. Understanding the evolution of change in volume of hippocampus and its individual subfields would provide unique insights into the relationship between volumetric reductions due to mTLE and the extent of impairments in motor sequence learning. Second, motor sequence learning was only related to initial motor memory acquisition in the current study. Future studies should examine other hippocampal-dependent tasks associated with other types of memory (e.g., non-motor sequence learning), which will provide greater insight into the role of specific sub-parts of the hippocampus. Third, we only investigated initial motor memory acquisition. Whether long-term memory or consolidation of the motor sequence is impacted by mTLE is an important consideration. Accordingly, future work should focus on how memories are sufficiently strengthened to be behaviorally salient, thus, allowing further insight into the role of specific hippocampal regions. Finally, since mTLE may be a heterogeneous group with varying hippocampal subfield anomalies difficult to identify solely based on MRI, future work can expand on the current findings by including a histopathology report according to the International League Against Epilepsy (ILAE) classification of Hippocampal sclerosis (Blümcke et al., 2013).

## Disclosure

We confirm that we have read the Journal’s position on issues involved in ethical publication and affirm that this report is consistent with those guidelines.

## Author Contributions

All authors reviewed and approved the manuscript content.

## Conflict of Interest Statement

The authors declare that the research was conducted in the absence of any commercial or financial relationships that could be construed as a potential conflict of interest.

## Footnotes

^1^https://surfer.nmr.mgh.harvard.edu/
